# Modality-specific improvements in sensory processing among baseball players

**DOI:** 10.1038/s41598-021-81852-x

**Published:** 2021-01-26

**Authors:** Koya Yamashiro, Yudai Yamazaki, Kanako Siiya, Koyuki Ikarashi, Yasuhiro Baba, Naofumi Otsuru, Hideaki Onishi, Daisuke Sato

**Affiliations:** 1 Institute for Human Movement and Medical Sciences, Niigata University of Health and Welfare, Niigata, 950-3198 Japan; 2 Department of Health and Sports, Niigata University of Health and Welfare, Niigata, 950-3198 Japan; 3 Department of Physical Therapy, Niigata University of Health and Welfare, Niigata, 950-3198 Japan

**Keywords:** Cognitive neuroscience, Motor control, Sensorimotor processing, Sensory processing

## Abstract

Long-term skills training is known to induce neuroplastic alterations, but it is still debated whether these changes are always modality-specific or can be supramodal components. To address this issue, we compared finger-targeted somatosensory-evoked and auditory-evoked potentials under both Go (response) and Nogo (response inhibition) conditions between 10 baseball players, who require fine hand/digit skills and response inhibition, to 12 matched track and field (T&F) athletes. Electroencephalograms were obtained at nine cortical electrode positions. Go potentials, Nogo potentials, and Go/Nogo reaction time (Go/Nogo RT) were measured during equiprobable somatosensory and auditory Go/Nogo paradigms. Nogo potentials were obtained by subtracting Go trial from Nogo trial responses. Somatosensory Go P100 latency and Go/Nogo RT were significantly shorter in the baseball group than the T&F group, while auditory Go N100 latency and Go/Nogo RT did not differ between groups. Additionally, somatosensory subtracted Nogo N2 latency was significantly shorter in the baseball group than the T&F group. Furthermore, there were significant positive correlations between somatosensory Go/Nogo RT and both Go P100 latency and subtracted Nogo N2 latency, but no significant correlations among auditory responses. We speculate that long-term skills training induce predominantly modality-specific neuroplastic changes that can improve both execution and response inhibition.

## Introduction

Long-term skills training induce neuroplastic changes in cortical areas associated with sensory, motor, and cognitive tasks. In short, it seems like that an athlete’s excellent performance largely depends on the dynamic functional and structural changes of the brain. Studies on athletes using somatosensory-evoked potentials (SEPs) and event-related potentials (ERPs) in response to tactile stimulation suggest that specific training can modify the activity of the somatosensory cortex and neuronal processing related to specific cognitive and inhibitory function^1–5^.

Our previous study investigated the effect of specific skills training on somatosensory information processing by comparing SEPs to stimulation of the dominant (right) hand index finger in a baseball player group and a general sports group (including track and field [T&F] athletes, soccer players, and swimmers) not requiring the same fine somatosensory discrimination or motor control of the hand as baseball players. The results revealed significant positive correlations between simple reaction time (SRT) and both peak P100 and peak N140 latencies exclusively in the baseball group, suggesting faster somatosensory information processing due to neuroplastic alterations associated with specific sensorimotor skills^1^. When hitting, baseball players are also required to stop bat movement as quickly as possible (e.g., if the incoming pitch is unhittable). This response requires active suppression of the motor commands associated with the bat swing. This inhibitory effect has been investigated in each sensory modality as Nogo N2, Nogo P3, and subtracted Nogo N2 using ERPs^6–12^. These studies showed that Nogo-related potentials were enhanced, delayed, and anteriorized in Nogo trials compared with Go trials in each sensory modality. Furthermore, these studies demonstrate that inhibitory effects exist in all sensory modalities to some extent. In other words, these indices of inhibition may be appropriate for assessing inhibitory functions in humans. Indeed, previous studies have shown that superior athletic skill requiring response inhibition is reflected by shorter visual Nogo N2 latency and enhanced Nogo N2^13^ and P3^14^ amplitudes and superior performance in Go/Nogo reaction time (Go/Nogo RT)^13–16^ in visual modalities. We also showed that the somatosensory subtracted Nogo N2 component was significantly shorter and larger in the baseball group than in the other sports group and there was a significant positive correlation between the Go/Nogo RT and both the latency and amplitude of subtracted Nogo N2 in the somatosensory modality. Reportedly, long-term training affected supramodal components and behavioral indices, and the Go/Nogo RT can be considered a good indicator of response inhibition ability in athletes such as baseball players, fencers, and boxers.

Similarly, Walther et al.^17^ reported modality-specific (visual versus auditory) inhibition in the frontoparietal network but not the prefrontal cortex using event-related magnetic resonance imaging (fMRI), suggesting both modality-specific and supramodal response inhibition networks. However, our previous experimental paradigms could not determine whether modality-specific or supramodal components contributed to the superior Go/Nogo RT in baseball players versus other athletes.

To more precisely distinguish between modality-specific and supramodal neuroplastic changes related to both execution and response inhibition, we compared dominant-hand somatosensory and auditory ERP components as well as Go/Nogo RTs of baseball players to T&F athletes. The supramodal components appear superimposed on the somatosensory and auditory modality-specific components, i.e., Nogo components include both modality-specific and supramodal components. However, considering that this study aimed to assess these components separately in two athlete groups, a subtraction method was employed to better distinguish the effects of response inhibition. In fact, Trope et al.^18^ reported activity specific to “no-go” trials using subtraction method. We hypothesized that if specific training alters sensory processing in neural circuits associated with the trained hand, Go/Nogo RT should be only faster for the somatosensory modality in the baseball group. Moreover, the amplitude of subtracted Nogo N2 may be larger across both modalities in baseball plays as a supramodal component.

## Methods

### Subjects

Twenty-two healthy male undergraduate university students participated in this study. Written informed consent was obtained from each subject after a full explanation of study objectives and methods. Ten subjects had played baseball for more than 9 years (baseball group) whereas the other twelve had performed T&F events for more than 7 years (T&F group). The baseball group and T&F group were matched for age (mean, 21.5 ± 0.7 years vs. 20.2 ± 0.7 years) and height (mean, 175.3 ± 3.9 cm vs. 173.4 ± 5.5 cm). The study was conducted in accordance with the Declaration of Helsinki and approved by the ethics committee of Niigata University of Health and Welfare, Niigata, Japan.

### Somatosensory and auditory stimulation

Somatosensory ERPs were elicited by constant current square-wave pulses (duration 0.2 ms) delivered to the second and fifth digits of the dominant hand by ring electrodes. The anode was placed at the distal interphalangeal joint and the cathode at the proximal interphalangeal joint of the corresponding digits. The fifth digit was stimulated for the Go condition and the second digit for the Nogo condition. Stimulus intensity at the fifth digit was fixed at three times the subject’s sensory threshold and that at the second digit was adjusted so that the subject felt the same sensation intensity as at the fifth digit^2^. These stimuli elicited no pain or other unpleasant sensations. Auditory ERPs were elicited binaurally by a pure tone delivered through headphones (60 dB sound pressure level, 50 ms duration, 10 ms rise time, and 10 ms fall time). A 1000 Hz pure tone was delivered for the Go condition and a 1500 Hz pure tone for the Nogo condition.

### Somatosensory and auditory Go/Nogo paradigms

Prior to the EEG, subjects had undergone five practice sessions of Go/Nogo paradigms (one session = 40 trials; total trials = 200) with a 1-min break between each of the somatosensory and auditory modalities to exclude the effect of short-term training. In the EEG sessions, subjects performed separate somatosensory and auditory Go/Nogo tasks in the same day, both consisting of 50 Go trials and 50 Nogo trials (i.e., equal 50% probabilities) presented in random order. All the subjects first performed the somatosensory task and then auditory task. Individual somatosensory stimuli were presented at 2-s inter-stimulus intervals (ISIs) and auditory stimuli at 1-s ISIs. Similar to the somatosensory modality, we conducted a preliminary experiment with the ISI set to 2 s in the auditory modality. However, the ISI at 2 s appeared to be longer in the auditory than in the somatosensory modality, which made the task more challenging. Therefore, the ISI was set to 1 s in the auditory modality. An ISI of approximately 1 s was used according with the previous Go/Nogo auditory paradigms^19,20^ to correct the difficulty of the task. In both somatosensory and auditory Go trials, subjects were instructed to press a button as fast as possible using the dominant second digit when they perceived the Go stimulus (current or sound).

### EEG recording

A SynAmps amplifier system and scan 4.3 software (Neuroscan, El Paso, TX, USA) were used for EEG acquisition. The EEG was recorded using nine scalp electrodes placed at Fz, Cz, Pz, F3, F4, C3, C4, P3, and P4 according to the 10–20 system. The left earlobe was used as a reference. Electrode impedance was maintained below 5 kΩ. EEG signals were recorded with a notch filter (50 Hz) at a sampling rate of 1000 Hz. Trials with responses exceeding ± 100 μV were excluded from averaging according to our previous studies^1,2^. The off-line band-pass filter was set at 0.5–60 Hz. In both the somatosensory and auditory Go/Nogo paradigms, 50 artifact-free Go and 50 artifact-free Nogo trials were averaged for each subject. Responses were analyzed from 100 ms before (baseline) to 500 ms after stimulus onset.

### Analyses

Stimulation elicited the P100 and N1 component positive and negative peaks around 100 ms in both somatosensory and auditory Go trials. To extract Nogo potentials, we subtracted the averaged waveforms of Go trials from Nogo trials according to previous studies^2,8,11,18,20^. The subtracted waveform exhibited a negative peak at 200 ms after somatosensory or auditory stimulus onset. This peak was termed the somatosensory or auditory subtracted Nogo N2 component, respectively. The peak amplitudes of P100, N1, and subtracted Nogo N2 were measured relative to pre-stimulus baseline. The peak latencies and amplitudes of P100, N1 and subtracted Nogo N2 were measured at frontal and midline electrodes (Fz, Cz, Pz, F3, and F4) between 60–120 ms and 120–250 ms after stimulus onset, respectively, as auditory N1^21,22^ and subtracted Nogo N2 are known to reach a maximum around the frontal region for both somatosensory^2^ and auditory^20^ modalities. The Shapiro–Wilk test was performed to confirm the normal distribution before analysis of variance (ANOVA). It was confirmed that all models were normally distributed. Latencies and amplitudes were compared by two-way ANOVA with five electrode positions (Fz, Cz, Pz, F3, F4) as a within-subject factor and group (baseball vs. T&F) as a between-subject factor. We performed Mauchly’s sphericity assumption test and if it was violated, the Greenhouse–Geisser epsilon was used to correct the degrees of freedom. Post hoc Bonferroni tests were performed to assess pair-wise differences in peak amplitude and latency of P100, N1 and subtracted Nogo N2 between groups and electrode positions. Differences in Go/Nogo RT and commission error rates were tested by Welch’s t-tests in both modalities. The Cohen’s d test also tested the effect size under between-subject factor (latency, Go/Nogo RT and comission error) compensate for the small sample size. Statistical significance was set at *p* < 0.05 for all tests. We also analyzed the bivariate correlations between Go/Nogo RT and both the latencies and amplitudes of P100, N1 and subtracted Nogo N2 at midline electrodes (Fz, Cz, Pz, F3, and F4).

## Results

### Modality-specific improvement in Go/Nogo RT among baseball players

The somatosensory Go/Nogo RT was significantly shorter in the baseball group than the T&F group (228.1 ± 25.3 ms vs. 252.0 ± 27.6 ms, t(19.793) = 2.100, *p* = 0.047, d = 0.84) while there was no group difference in auditory Go/Nogo RT (249.2 ± 25.2 ms vs. 248.5 ± 27.6 ms, t(17.334) = 0.071, *p* = 0.943, d = 0.03). The commission error rates did not differ between groups for either somatosensory (0.55 ± 0.89% vs 1.60 ± 1.97%, t(15.9) = 1.652, *p* = 0.118, d = 0.67) or auditory (1.49 ± 1.49% vs 2.08 ± 2.34%, t(18.862) = 0.715, *p* = 0.483, d = 0.29) modalities, respectively.

### Somatosensory-specific changes in both Go and subtracted Nogo N2 component

**Figure 1:**
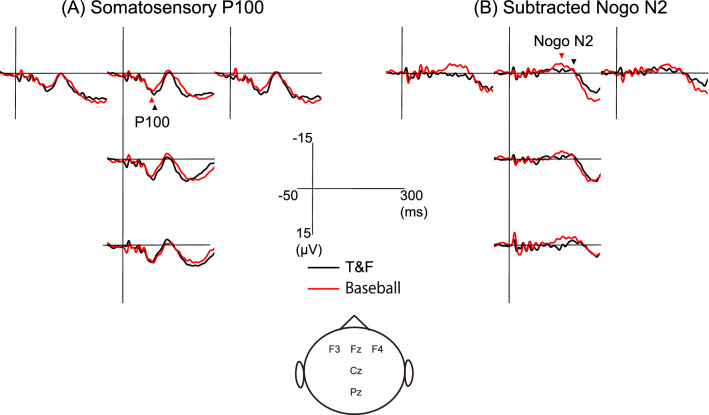
Grand averaged somatosensory P100 and subtracted Nogo N2. Grand averaged somatosensory P100 for Go trials (**A**) and subtracted Nogo N2 (**B**) in the T&F and baseball groups. The black and red triangles show the peak latency of each component in the T&F and baseball groups, respectively.

Figure 1A shows the grand averaged waveforms in somatosensory Go trials at five electrode positions for the baseball and T&F groups, and Fig. 1B presents the grand averaged subtracted Nogo N2 at each electrode position for both groups obtained by subtracting the grand averaged Go response from the grand averaged Nogo response. The peak latencies and amplitudes of P100 and subtracted Nogo N2 at the five frontal and midline electrode sites Fz, Cz, Pz, F3, and F4 are summarized for both groups in Table 1. Two-way ANOVA revealed a significant main effect of electrode position (F_(2.417, 48.345)_ = 3.030, *p* = 0.048 ε = 0.604, *η*^2^ = 0.132) and group (F_(1, 20)_ = 6.286, *p* = 0.021, *η*^2^ = 0.239, d = 0.67) on the P100 latency but no group × electrode position interaction. The P100 latency was significantly shorter at Cz than F3 and in the baseball group than the T&F group. Two-way ANOVA also revealed a significant main effect of group on subtracted Nogo N2 latency (F_(1, 20)_ = 6.838, *p* = 0.017, *η*^2^ = 0.255, d = 1.10) but no main effect of electrode position or group × electrode position interaction. The subtracted Nogo N2 peak latency was significantly shorter in the baseball group than the T&F group. On the other hand, there was no significant main effects on P100 and subtracted Nogo N2 amplitudes.

**Table 1:** The peak latency and amplitude ± S.D of somatosensory Go P100 and subtracted Nogo N2.

Electrode	Latency (ms)				Amplitude (µV)			
	Go P100		Subtracted Nogo N2		Go P100		Subtracted Nogo N2	
	T&F	Baseball*	T&F	Baseball*	T&F	Baseball	T&F	Baseball
Fz	102 ± 9	91 ± 9	199 ± 26	174 ± 26	7.4 ± 3.8	6.4 ± 2.6	− 3.9 ± 2.6	− 4.9 ± 2.6
Cz	96 ± 8	94 ± 7	202 ± 23	175 ± 25	7.2 ± 3.5	6.5 ± 3.1	− 3.9 ± 2.3	− 4.7 ± 2.3
Pz	100 ± 13	99 ± 11	204 ± 18	177 ± 27	6.4 ± 3.1	6.4 ± 3.1	− 3.2 ± 2.7	− 4.9 ± 3.1
F3	105 ± 11	97 ± 10	199 ± 27	176 ± 25	6.7 ± 3.1	6.1 ± 2.6	− 2.4 ± 2.3	− 4.3 ± 2.4
F4	108 ± 10	97 ± 9	199 ± 28	170 ± 19	7.9 ± 2.9	6.5 ± 2.0	− 3.6 ± 2.6	− 4.3 ± 2.2

The asterisk shows a significant groups difference.

*p < 0.05.

### Absence of changes in auditory ERP component

**Figure 2:**
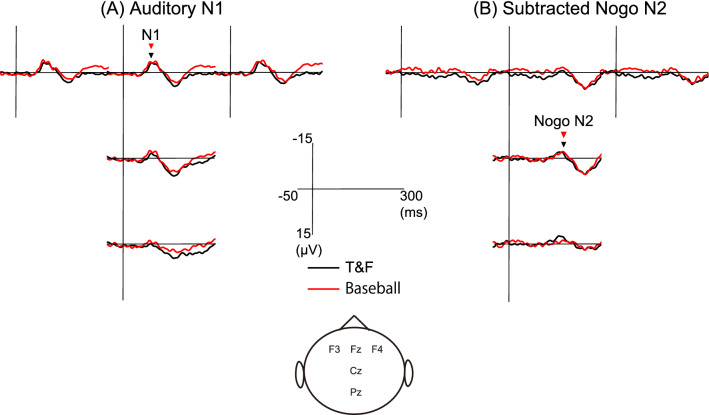
Grand averaged auditory N1 and subtracted Nogo N2. Grand averaged auditory N1 for Go trials (**A**) and subtracted Nogo potentials (**B**) in the T&F and baseball groups. The black and red triangles show the peak latency of each component in the T&F and baseball groups, respectively.

Figure 2A shows the grand averaged waveforms at five electrode positions in auditory Go trials for the baseball and T&F groups, and Fig. 2B presents the grand averaged subtracted Nogo N2 for each electrode position and both groups obtained as described for the somatosensory Go/Nogo sessions. The peak latencies and amplitudes of N1 and subtracted Nogo N2 at the five electrode sites for both groups are presented in Table 2. Two-way ANOVA revealed no significant main effect of electrode or group and no group × electrode position interaction on the latency of N1 and subtracted Nogo N2. On the other hand, two-way ANOVA revealed a significant main effect of electrode position on N1 amplitude (F_(2.007, 40.141)_ = 28.383, *p* < 0.001, *η*^2^ = 0.587) but no main effect of group and no group × electrode position interaction. The N1 amplitude was significantly larger at frontal electrode positions (F3, Fz, and F4) than Cz and Pz.

**Table 2:** The peak latency and amplitude ± S.D of auditory Go N1 and subtracted Nogo N2.

Electrode	Latency (ms)				Amplitude (µV)			
	Go N1		Subtracted Nogo N2		Go N1		Subtracted Nogo N2	
	T&F	Baseball	T&F	Baseball	T&F	Baseball	T&F	Baseball
Fz	98 ± 8	97 ± 10	176 ± 12	171 ± 15	− 3.9 ± 2.6	− 4.6 ± 2.6	− 1.7 ± 3.7	− 3.0 ± 2.6
Cz	96 ± 9	97 ± 9	173 ± 14	166 ± 14	− 2.5 ± 2.7	− 3.4 ± 2.3	− 3.0 ± 2.9	− 3.1 ± 2.2
Pz	97 ± 11	96 ± 14	179 ± 15	174 ± 20	− 1.0 ± 2.4	− 2.1 ± 2.0	− 3.7 ± 2.2	− 2.6 ± 2.0
F3	98 ± 8	94 ± 10	175 ± 14	176 ± 14	− 3.7 ± 2.6	− 4.9 ± 2.5	− 0.8 ± 3.0	− 3.0 ± 3.3
F4	99 ± 12	99 ± 10	176 ± 18	174 ± 18	− 4.3 ± 2.7	− 5.0 ± 2.8	− 1.6 ± 4.0	− 3.2 ± 1.5

### Associations of somatosensory Go/Nogo RT with somatosensory P100 and subtracted Nogo N2 components

**Figure 3:**
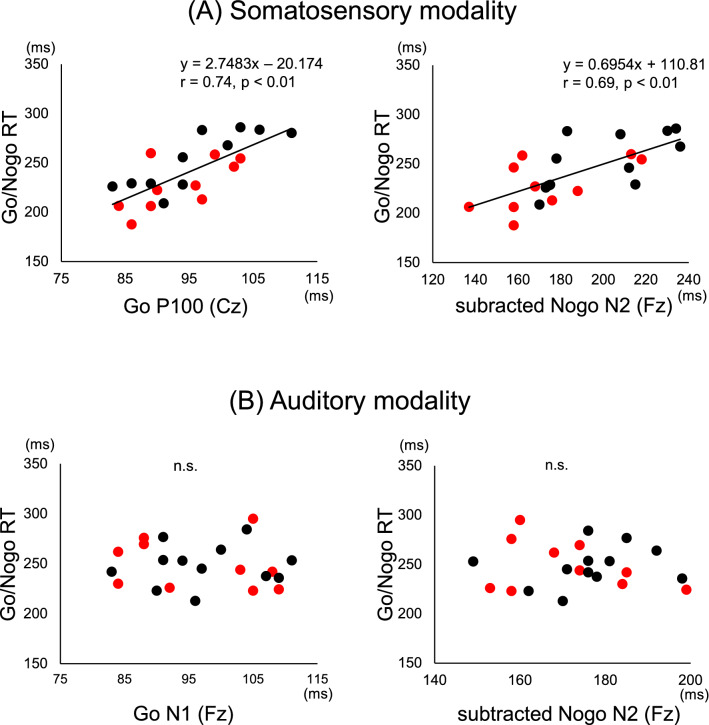
Correlations in somatosensory and auditory modality. Correlations between Go/Nogo RT and the latency of Go P100 and N1 as well as the subtracted Nogo N2 components in (**A**) the somatosensory modality and (**B**) the auditory modality. The black and red circles show the T&F and baseball groups, respectively.

**Figure 4:**
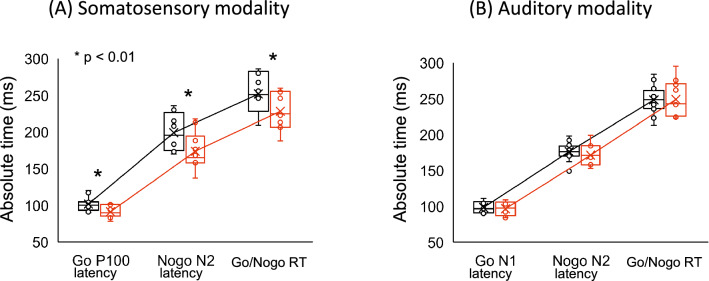
Comparison of neurophysiological and behavioral indices in T&F and baseball group. The box plots represent the mean (± SD) of the absolute time (ms) of neurophysiological and behavioral indices in (**A**) the somatosensory modality and (**B**) the auditory modality. The T&F and baseball groups are represented by black and red boxes, respectively. The minimum score is the lowest score, excluding outliers (shown at the end of the lower whisker). The maximum score is the highest score, excluding outliers (shown at the end of the upper whisker). The median, i.e., the midpoint of the data, is shown by the line that divides the box into two parts. The lower (Q1) and upper (Q3) quartiles are the 25% of scores that fall below the lower quartile value and 75% of the scores that fall below the upper quartile value, respectively. The interquartile range, i.e., the box, shows the middle 50% of scores. The upper and lower whiskers represent scores outside the middle 50% (i.e., the upper and lower 25% of scores). An outlier is judged by whether it falls within Q_1_ − (IQR × 1.5) − Q_3_ + (IQR × 1.5).

Correlation analysis across all subjects revealed a significant positive association between somatosensory Go/Nogo RT and P100 latency at the Fz and Cz electrodes, and between Go/Nogo RT and subtracted Nogo N2 latency at Fz, Cz, Pz, F3 and F4 electrodes (Fig. 3A, Table 3). Alternatively, there were no significant correlations of Go/Nogo RT with P100 and subtracted Nogo N2 peak amplitudes in the somatosensory modality. Figure 4A shows comparison of neurophysiological and behavioral indices in the somatosensory modality.

**Table 3:** The r values of correlations between Go/Nogo RT and the latencies of modality specific components and supramodal components at the five electrodes in the somatosensory and auditory modalities.

	Fz	Cz	Pz	F3	F4
**Somatosensory**					
Go P100	0.58**	0.74**	0.32	0.32	0.26
Subtracted Nogo N2	0.69**	0.61**	0.59**	0.73**	0.67**
**Auditory**					
Go N1	− 0.07	0.16	0.16	0.08	− 0.01
Subtracted Nogo N2	− 0.07	− 0.15	− 0.14	− 0.19	− 0.41

**p < 0.01.

### Associations of auditory Go/Nogo RT with auditory N100 and subtracted Nogo N2 components

Correlation analysis across all subjects revealed no significant associations between auditory Go/Nogo RT and either N1 latency or subtracted Nogo N2 latency (Fig. 3B, Table 3). There were also no significant correlation between Go/Nogo RT and either N1 or subtracted Nogo N2 peak amplitudes in the auditory modality. Figure 4B shows comparison of neurophysiological and behavioral indices in the auditory modality.

## Discussion

In the present study, we compared finger-targeted somatosensory ERP components and auditory ERP components between baseball players and age-matched T&F athletes to examine if long-term skills training induces modality-specific or supramodal changes in sensory processing. The results showed that (1) somatosensory P100 latency, subtracted Nogo N2 latency, and Go/Nogo RT were significantly shorter in the baseball group than the T&F group, while there were no group differences in auditory N1 latency, subtracted Nogo N2 latency, and Go/Nogo RT, and (2) there were significant positive correlations between Go/Nogo RT and somatosensory P100 latency and subtracted Nogo N2 latency but not between corresponding auditory ERP components. These findings suggest that the repetitive grasping movements employed by baseball players for batting, throwing, and catching as well as the response inhibition required for batting primarily facilitate hand/digit somatosensory processing by inducing neuroplastic alterations in relevant circuits that are reflected by modality-specific changes in SEP components and Go/Nogo RT.

### Improved modality-specific sensory processing among baseball players

The baseball group demonstrated significant reductions in somatosensory P100 latency and Go/Nogo RT but not in auditory N1 latency and Go/Nogo RT compared to the T&F group. Neuroplastic alterations in the somatosensory cortex have been demonstrated in athletes and other professionals requiring extensive specific sensorimotor skills training^1–5^ that are reflected by shorter-latency SEP and ERP components. Peak latencies are important indices of stimulus classification speed and/or stimulus evaluation time in SRT^1^ and Go/Nogo paradigms^2^. These findings support the notion that the somatosensory P100 latency following digit stimulation is shorter in baseball players than T&F athletes because the latter group does not require the same degree of fine somatosensory discrimination or motor skill in the hand. Human and animal studies have described a specific cortical grasping network^23,24^ that includes hand somatosensory processing circuits^25^. Therefore, long-term baseball training but not T&F training may specifically facilitate somatosensory processing and discrimination within this circuit as reflected by reduced P100 latency and Go/Nogo RT. On the other hand, there were no significant reductions in auditory N1 latency and Go/Nogo RT among the baseball group as this modality is not preferentially engaged by baseball or T&F training. However, a recent study using the oddball paradigm reported that auditory N2 and P3 latencies were shorter in athletes such as fastball sports players and dancers than in controls. The authors speculated that auditory processing is especially vital in competitive fastball sports and performing arts like dance, and thus intense training may activate and strengthen networks responsible from auditory-related cognition through use-dependent neuroplastic processes^26^. We propose two possible explanations for this apparent discrepancy. First, both groups in the present study were athletes (baseball and T&F) and we did not include an untrained group. Thus, both groups may have incurred similar neuroplastic changes in the auditory cortex resulting in uniformly better auditory discrimination processing than untrained controls. The second possibility is that ERP components are differentially sensitive to training-induced changes. The shorter-latency N1 component primarily reflects lower-order sensory transmission^27^ while the longer latency N2 (and P3) reflect higher-order cognitive processes more sensitive to training-induced plasticity (and thus reduced latency). Moreover, there was also a significant positive correlation between somatosensory Go P100 latency and Go/Nogo RT but not between auditory Go N1 latency and Go/Nogo RT. Therefore, somatosensory Go P100 and auditory Go N1 may reflect different processing functions, indicating a training-dependent neuroplasticity change only somatosensory Go P100. Indeed, several studies have reported structural and functional plasticity in the somatosensory cortex of musicians^28,29^. The auditory N1 amplitude was significantly larger at frontal electrode positions (F3, Fz, and F4 than Cz and Pz) independently of the group, which is consistent with previous studies^21,22^.

### Improved somatosensory response inhibition among baseball players

The visual Nogo N2 latency is known to reflect inhibition of visually-evoked response execution^30^, and we found a significant reduction in somatosensory subtracted Nogo N2 latency among the baseball group compared to the T&F group, consistent with previous studies^2^. On the other hand, there was no group difference in auditory subtracted Nogo N2 latency. Several studies have reported that visual Go/Nogo RT is shorter in fencers ^13,31^. For example, Russo et al. found that the visual ERPs recorded in posterior cingulate gyrus, which is associated with visual stimulus discrimination, started earlier and were larger in fencers than non-fencers^31^, while Zhang et al. found that visual N2 latency was shorter in experienced fencers, who like baseball players require rapid response inhibition, compared to non-fencers^13^. Thus, superior athletic skill requiring response inhibition can be reflected by shorter visual Nogo N2 latency. Interestingly, Akatsuka et al.^32^ used the somatosensory Go/Nogo paradigm after acute exercise. They found that moderate exercise affected the amplitude of Nogo-N140 and significantly enhanced the peak amplitude of Nogo-N140 in Fz and Cz, suggesting that Nogo-N140 is modulated by acute aerobic exercise. This research shows that repetition of exercise can be a sufficient stimulus to modulate the somatosensory component (i.e., subtracted Nogo N2).

On the other hand, other studies report conflicting results. For example, Bruno et al.^33^ found that in a visual Go/Nogo task, long-term limb immobilization can modulate inhibitory ERP responses, suggesting that inhibition-related EEG activity is significantly reduced not only by the presence of a cast over the limb but also by the duration of the immobilization. These findings indicate that the manner of use affects inhibitory brain activity. To the best of our knowledge, the present study is the first to report a reduction in somatosensory subtracted Nogo N2 latency only without any change to auditory subtracted Nogo N2. In accord with these results, Nakata et al.^34^ found shorter Go/Nogo RT and Nogo N2 latency in a visual Go/Nogo paradigm but no similar association between Nogo N2 latency and Go/Nogo RT in an auditory Go/Nogo paradigm. Since there was a significant correlation between behavioral and neurophysiological indices in both the baseball and T&F groups, we speculate that neuroplasticity may be more likely to occur in the tactile modality than in the auditory modality. Therefore, our findings may reflect a characteristic difference between somatosensory and auditory subtracted Nogo N2. Thus, further studies should compare somatosensory and visual subtracted Nogo N2 latencies in appropriate training groups as a measure of modality specificity.

### Relationship between subtracted Nogo N2 amplitude and Go/Nogo RT in somatosensory and auditory modalities

Our previous study showed that somatosensory subtracted Nogo N2 amplitude was larger in baseball players than a general sports group and further that there was a significant correlation between subtracted Nogo N2 amplitude and Go/Nogo RT in both groups. In contrast, no significant enhancement of subtracted Nogo N2 amplitude was found in the baseball group compared to the T&F group and there was no correlation between subtracted Nogo N2 amplitude and Go/Nogo RT in either group. A reasonable explanation for these discrepancies is the equal probability Go/Nogo paradigm used in the current study (50% Go/50% Nogo) to eliminate the stimulus probability effect. Our previous study adopted 25% Nogo probability and Nakata et al. found that a decrease in Nogo probability increased the peak Nogo N2 amplitude^8^. Nakata et al.^35^ also showed the Nogo N140 (analogous to subtracted Nogo N2 in the somatosensory modality) was not significantly associated with Go/Nogo RT. Therefore, subtracted Nogo N2 amplitude would have relatively less effect on Go/Nogo RT using the current equiprobability Go/Nogo paradigm. We also found no significant correlation between auditory subtracted Nogo N2 amplitude and Go/Nogo RT, consistent with previous auditory Go/Nogo studies. For instance, Falkenstein et al. found an enhancement of Nogo N2 amplitude only after visual stimuli in the audio-visual Go/Nogo paradigm^36^. Smith et al.^37^ found that changes in specific auditory ERP components of the cued-Go/Nogo paradigm differed among subjects according to reaction time (i.e., basal processing skill). While a marginal enhancement of Nogo N2 amplitude was observed, it was not significantly larger in the fast group, while the Nogo P3 effect was larger in the fast RT group. The authors suggested that the inhibitory process may be reflected by the enhancement of Nogo P3 amplitude rather than the enhancement of Nogo N2 amplitude in the auditory modality. Taken together, Nogo N2 amplitude is likely unrelated to Go/Nogo RT in the equiprobable somatosensory and auditory Go/Nogo paradigms.

### Limitations of the study

We acknowledge that there are two major limitations to this study. Firstly, the number of subjects was small: the baseball group had 10 subjects and the T&F group had 12. It was extremely difficult to recruit a larger number of subjects considering that the subjects were required to have similar experience, skill level, and physical characteristics in each sport. Secondly, a control group with no experience of the training was not used. The present study compared athletes from different sports, i.e., the effect of variations in the types of exercise was examined. However, participants in both groups were regularly exercising individuals. As we did not include a “no-exercise” control group, we cannot judge differences between athletes and nonathletes.

## Conclusion

The present results show that baseball players demonstrate superior somatosensory processing and discrimination of the digits as measured by the Go/Nogo paradigm but no difference in auditory discrimination compared to T&F athletes not requiring find hand skills. Thus, skills training-dependent improvements in sensory processing are primarily modality-specific. Further, these findings suggest that somatosensory P100 latency is the main contributor to improved Go/Nogo RT in the somatosensory Go/Nogo paradigm, while subtracted Nogo N2 amplitude has little effect.
